# USP29 activation mediated by FUBP1 promotes AURKB stability and oncogenic functions in gastric cancer

**DOI:** 10.1186/s12935-024-03224-5

**Published:** 2024-01-17

**Authors:** Rongfu Tu, Ye Kang, Yiwen Pan, Yanyan Da, Doudou Ren, Ru Zhang, Zeqiong Cai, Yijia Liu, Jiao Xu, Junpeng Ma, Zhiyong Zhou, Shupeng Yin, Xiaozhuang Li, Peng Zhang, Qi Zhang, Jingchao Wang, Xinlan Lu, Chengsheng Zhang

**Affiliations:** 1 Department of Cancer Precision Medicine, The MED-X Institute, The First Affiliated Hospital of Xi’an Jiaotong University, Building 21, Xi’an, China; 2 Precision Medicine Center, The First Affiliated Hospital of Xi’an Jiaotong University, 277 Yanta West Road, Xi’an, 710061 China; 3 Center for Molecular Diagnosis and Precision Medicine, The First Affiliated Hospital, Jiangxi Medical College, Nanchang University, 1519 Dongyue Dadao, Nanchang, 330209 China; 4 Department of Clinical Laboratory, The First Affiliated Hospital, Jiangxi Medical College, Nanchang University, 17 Yongwai Zhengjie, Nanchang, 330006 China; 5 Jiangxi Provincial Center for Advanced Diagnostic Technology and Precision Medicine, The First Affiliated Hospital, Jiangxi Medical College, Nanchang University, 1519 Dongyue Dadao, Nanchang, 330209 China; 6 Department of Medical Genetics, The First Affiliated Hospital, Jiangxi Medical College, Nanchang University, 1519 DongYue Dadao, Nanchang, 330209 China; 7 Department of Emergency Surgery, Union Hospital, Tongji Medical College, Huazhong University of Science and Technology, Wuhan, China; 8 Guangdong Key Laboratory of Genome Instability and Human Disease Prevention, Department of Biochemistry and Molecular Biology, Shenzhen University School of Medicine, Shenzhen, 518055 China; 9 Department of Gastroenterology, The First Affiliated Hospital of Xi’an Jiaotong University, Xi’an, Shaanxi 710061 China

**Keywords:** Gastric Cancer, USP29, AURKB, FUBP1, Targeted therapy

## Abstract

**Background:**

Gastric cancer is a highly prevalent cancer type and the underlying molecular mechanisms are not fully understood. Ubiquitin-specific peptidase (USP) 29 has been suggested to regulate cell fate in several types of cancer, but its potential role in gastric carcinogenesis remains unclear.

**Methods:**

The expression of USP29 in normal and gastric cancer tissues was analyzed by bioinformatics analysis, immunohistochemistry and immunoblot. Gene overexpression, CRISPR-Cas9 technology, RNAi, and *Usp29* knockout mice were used to investigate the roles of USP29 in cell culture, xenograft, and benzo[a]pyrene (BaP)-induced gastric carcinogenesis models. We then delineated the underlying mechanisms using mass spectrometry, co-immunoprecipitation (Co-IP), immunoblot, ubiquitination assay, chromatin immunoprecipitation (ChIP), quantitative real-time PCR (qRT-PCR), and luciferase assays.

**Results:**

In this study, we found that USP29 expression was significantly upregulated in gastric cancers and associated with poor patient survival. Ectopic expression of USP29 promoted, while depletion suppressed the tumor growth in vitro and in vivo mouse model. Mechanistically, transcription factor far upstream element binding protein 1 (FUBP1) directly activates USP29 gene transcription, which then interacts with and stabilizes aurora kinase B (AURKB) by suppressing K48-linked polyubiquitination, constituting a FUBP1-USP29-AURKB regulatory axis that medicates the oncogenic role of USP29. Importantly, systemic knockout of *Usp29* in mice not only significantly decreased the BaP-induced carcinogenesis but also suppressed the Aurkb level in forestomach tissues.

**Conclusions:**

These findings uncovered a novel FUBP1-USP29-AURKB regulatory axis that may play important roles in gastric carcinogenesis and tumor progression, and suggested that USP29 may become a promising drug target for cancer therapy.

**Supplementary Information:**

The online version contains supplementary material available at 10.1186/s12935-024-03224-5.

## Background

Gastric cancer is the fifth most common malignancy and the third leading cause of cancer death globally [1]. Despite tremendous efforts made to study gastric cancer, the key molecular mechanisms that drive gastric carcinogenesis and tumor progression are not fully understood [2, 3]. AURKB is a serine/threonine protein kinase that belongs to the aurora kinase family along with aurora kinase A (AURKA) and aurora kinase C (AURKC) [4]. AURKB-mediated histone H3 phosphorylation is essential for chromosome segregation during cell division, and AURKB deregulation triggers aneuploidy and genomic instability, which drives carcinogenesis [5]. Moreover, AURKB is involved in regulating protein stability/activity of several oncogenic drivers or tumor suppressors, including c-MYC [6], Snail1 [7] and p53 [8]. Deregulation of AURKB was observed in a variety of tumors [4], including gastric cancer [9], and its overexpression was associated with tumor progression [9, 10] and drug resistance [11, 12]. However, the upstream regulatory mechanisms underlying the AURKB oncogenic activity in cancer cells remain poorly understood.

Recent studies indicated that posttranslational modification of AURKB plays a key role in maintaining its oncogenic role. AKT was reported to phosphorylate AURKB on threonine 73, which protected it from proteasome degradation in cancer cells, and in PI3K/PTEN pathway altered tumor cell lines that lack a co-existence of KRAS-BRAF mutation are highly addicted to AKT maintenance of AURKB [13]. Similarly, BOP1 ribosomal biogenesis factor (BOP1) was reported to promote the malignancy of colorectal cancer cells by inducing higher pAURKB at Thr 232 (active form of AURKB) [14]. Ubiquitination-induced degradation of AURKB is critical to controlling its functions as a chromosomal passenger complex protein kinase [15, 16]. Protein ubiquitination is a highly dynamic process and can be reversed by deubiquitylating enzyme [17], and deubiquitylating enzymes ubiquitin-specific peptidase (USP) 35 [18], USP13 [19] and USP48 [20] were reported to modulate AURKB stability during cell cycle progression.

The potential role of USP29 in gastric cancer remains largely unknown, while USP29 was reported to stabilize Snail1 and promote the migration of gastric cancer cells in vitro [21]. We previously reported that USP29 stabilizes oncogenic MYC (including c-MYC and N-MYC) and HIF1α in normoxia and hypoxia, respectively, thereby promoting tumor metabolism and progression in c-MYC driven B cell lymphoma and N-MYC driven neuroblastoma [22]. Consistently, USP29 was also reported to stabilize HIF1α in hepatocellular carcinoma by another group [23]. In triple-negative breast cancer, USP29 was recently reported to be phosphorated and activated by cyclin-dependent kinase 1 (CDK1), and then deubiquitinate and stabilize twist family bHLH transcription factor 1 (TWIST1), which is a key regulator of epithelial-mesenchymal transition [24]. Moreover, USP29 was reported to deubiquitinate and stabilize cell division cycle 25 A (CDC25A) [25].

Here we observed that USP29 was overexpressed in gastric cancers and associated with poor patient outcomes. In terms of mechanism, *USP29* is transcriptionally activated by FUBP1, and the deregulated USP29 interacts with and stabilizes AURKB, forming a FUBP1-USP29-AURKB axis that enables tumor cell proliferation and survival in gastric cancers. Importantly, deletion of *Usp29* suppressed the mice forestomach tumor formation induced by BaP and Aurkb expression in the tissues. This study revealed an underlying molecular mechanism involving the oncogenic role of USP29, and identified a potential therapeutic target for gastric cancer.

## Materials and methods

### Cell culture

Cell lines HGC-27, SNU-216, HCT116, A549, SKN-BE2 and 293T were obtained from Procell Life Science & Technology Company (Wuhan, China), MGC-803 cells were obtained from American Type Culture Collection (Manassas, USA), all cell lines were authenticated by short tandem repeat (STR) analysis. MGC803, A549, HCT116 and 293T cells were grown in DMEM (Hyclone, USA) medium containing 10% fetal bovine serum (FBS, Zeta Life, USA) and 1% penicillin/streptomycin (Hyclone, USA). HGC-27, SNU-216 and SKN-BE2 cells were cultured in RPMI-1640 medium (BI, Israel) supplemented with 10% FBS and 1% penicillin/streptomycin. The cells were also tested negative for mycoplasma using PCR.

### Tumor samples

The gastric cancer specimens and the adjacent tissues were obtained from the patients who had not received chemotherapy or radiotherapy prior to the operation at The First Affiliated Hospital of Xi’an Jiaotong University, and confirmed by histopathological examinations. The protocols for human studies were approved by the Ethics Committee of The First Affiliated Hospital of Xi’an Jiaotong University.

### Antibodies and reagents

Primary antibodies for USP29 (Cat# AP2153c, Abcepta), AURKB (Cat# 3094, CST), FUBP1 (Cat# A5587, Abclonal), Flag-tag (Cat# 20543-1-AP, Proteintech), HA-tag (Cat# AE008, Abclonal), Myc-tag (Cat# AE009, Abclonal), ACTIN (Cat# 66009-1-Ig, Proteintech) were purchased from indicated companies. Cycloheximide (CHX; Cat# 01810) was obtained from Sigma Aldrich.

### Virus production and infection

For virus production, 293T cells were transfected with the lentiviral vector-derived plasmids (pLKO.1 for shRNA, lentiCRISPRv2 for sgRNA, and pHAGE for overexpression) and the helper plasmids (pMD2.G and psPAX2) using Lipo 2000 (Thermo Fisher Scientific). Viral supernatants were collected at 48 h post transfection. After passing through 0.22 μm filters, an appropriate amount of viruses was used to infect target cells in the presence of 5 µg/mL polybrene (sc-134,220, Santa Cruz). After incubation for additional one day, the infected cells were selected by puromycin (2–5 µg/ml) for at least 36 h before additional experiments were performed.

### Cell proliferation assay

Cells were plated in 96-well plates (1,000 cells per well with triplicates) in 100 µL growth medium and cultured for 24 h. At indicated time points, cells were replaced with 90 µL fresh growth medium supplemented with 10 µL Cell Counting Kit-8 (CCK-8) reagents (Cat# C0005, TargetMol, USA) followed by incubation at 37 °C for 1.5 h. The absorbance optical density (OD) value was measured at 450 nm using a KHB ST-360 plate reader.

### Immunohistochemistry (IHC)

The tissue microarrays of human gastric cancer were purchased from Bioaitech (Xi’an, China). The tissues were stained with anti-FUBP1 and anti-USP29 antibodies, respectively. The IHC scores were measured as previously published [26]. In brief, each sample was scored based on the staining intensity: negative staining was 0, weak positive was 1, positive was 2, and strong positive was 3.

### Immunoblotting

Cells were collected in the lysis buffer (50 mM Tris-HCl, pH 8.0, 150 mM NaCl, 1.1% NP-40, 1% SDS) supplemented with protease inhibitor cocktail (bimake, USA). The protein concentrations of lysates were determined using BCA method (Thermo Fisher Scientific, USA). Equal amount of total proteins were resolved by SDS polyacrylamide gel (SDS-PAGE). Separated proteins were transferred onto the PVDF membranes (Merck Millipore, USA); blots were soaked in 5% non-fat milk in Tris-buffered saline (TBS) containing 0.1% Tween 20 (TBST) for 1 h at room temperature and then incubated with primary antibodies at 4 °C overnight. After washing three times in TBST, blots were incubated with appropriate horseradish peroxidase-conjugated secondary antibodies (1:5,000) diluted in TBST for 1 h at room temperature followed by 3 washes with TBST. Immunoreactivity was detected with SuperSignal Chemiluminescent Substrate (Bio-Rad, USA) and visualized using AMERSHAM Imagequant 800 imaging System.

### RNA isolation and quantitative real-time PCR (qRT-PCR)

Total cellular RNA was extracted with Trizol reagent following the manufacturer’s instruction. Complementary DNA was reverse transcribed from 1 µg RNA with the ReverTra Ace qPCR RT kit (Vazyme, China). Quantitative PCR was performed using FAST SYBR Green Master Mix on CFX Connect Real-Time PCR System (GenStar, China). Relative expression of the mRNA was calculated by 2^−ΔΔCt^ method and normalized to ACTIN using specific primers as shown in Supplementary Table S1.

### Mass spectrometry analysis

Total proteins extracted from MGC-803 cells with or without overexpression of Flag-USP29were subjected to immunoprecipitation using antibodies against Flag-tag. The associated proteins were resolved by SDS-PAGE and gel slices including all proteins were excised for mass spectrometry analysis. LC-MS/MS analysis was performed in Beijing Qinglian Biotech Company as previously published [27]. For data analysis, candidates with coverage of less than 30% were recognized as low abundance of protein and removed.

### Immunoprecipitation (IP)

Cells were lysed in 600 µL NP40 buffer (50 mM Tris-HCl, pH 7.4, 150 mM NaCl, 1% NP-40, 1 mM EDTA, 5% glycerol) supplemented with 1× protease inhibitor cocktail on ice for 30 min. The lysates were centrifuged (12,000×g for 30 min) at 4 °C and the cell debris was discarded. 10% of the supernatant was saved as input to detect protein expression, and the remaining cell extract was incubated with 1 µg indicated antibody at 4 °C for 4 h, followed by the addition of protein A/G-agarose beads (Cat# P2055, Beyotime). The beads were washed and the IP product was obtained with 60 µL of 2x SDS-PAGE loading buffer incubated at 99 °C for 10 min, followed by western blotting analysis using the respective antibody.

### Chromatin immunoprecipitation (ChIP)

ChIP was performed as previously described [22]. Briefly, gastric cancer cells cultured in 10 cm dish (grown to 80%) were fixed with 1% formaldehyde for 10 min, quenched with 0.125 M glycine for 5 min at 37 °C, and then lysed in SDS lysis buffer (1%SDS, 10mM EDTA, 50mM Tris-HCl, pH8.1). Cell lysate was sonicated by shear chromatin DNA to a size range of 300–800 bp. The supernatant was diluted with 900 µL ChIP dilution buffer (1%Triton X-100, 2mM EDTA, 150mM NaCl, 20mM Tris-HCl, pH 8.1) and precleared with IgG and 60 µl agarose beads for 1 h. The supernatant fraction was immunoprecipitated with indicated antibodies (2 µg) against FUBP1 overnight at 4 °C. Antibody-chromatin complexes were pulled down with protein A/G agarose for 40 min at 4 °C. De-crosslinked DNA was subjected to qPCR analysis using specific primers listed in Supplementary Table S1.

### Time-course analysis of AURKB degradation

Cycloheximide (CHX) pulse-chase experiments were conducted to determine the half-life of AURKB protein. Cells were treated with CHX (100 µg/mL) and then harvested at specific time-points. Total cell lysates were separated by SDS-PAGE and protein levels were analyzed by immunoblot. AURKB protein band densities were quantified by Image lab software and normalized to ACTIN.

### Ubiquitination analysis

Cells were lysed in 100 µL SDS lysis buffer (50 mM Tris-HCl, pH 7.4, 150 mM NaCl, 1% NP-40, 1% SDS, 1 mM EDTA). Cell lysates were denatured at 95 °C for 10 min to disrupt protein interaction, and then diluted with 900 µL NP40 buffer (50 mM Tris-HCl, pH 7.4, 150 mM NaCl, 1% NP-40, 1 mM EDTA), and subjected to centrifugation at 12,000 g for 15 min. 10% of supernatant was saved as input to detect protein expression, and the remaining cell extract underwent immunoprecipitation with specific antibodies, followed by immunoblot analysis of protein polyubiquitination.

### Xenograft model

For xenografts, female BALB/c nude mice (6 weeks old, ordered from Gempharmatech Co., Ltd) were injected subcutaneously with 2 million MGC-803 cells diluted in 100 µl PBS). Tumor volumes were measured every 2 days and tumor weights were assessed in sacrificed animals. All mice were maintained in specific pathogen free animal facility of Laboratory Animal Center of Xi’an Jiaotong University. All animal experiments were performed with approval from the Institutional Animal Care and Use Committee of Xi’an Jiaotong University.

### Benzo[a]pyrene (BaP)-induced gastric cancer

Wildtype and *Usp29*^*−/−*^ mice in mixed genetic background (129/B6) were obtained from Dr. Guoliang Qing (Medical research institute, Wuhan University), the mice information and genotyping analysis were described previously [28]. In order to induce forestomach carcinogenesis, 6–8 weeks old mice were treated with 50 mg/kg BaP (Med Chem Express, HY-107,377) by intragastric administration twice a week for 6 weeks, all mice were sacrificed at 17 weeks after the last week of treatment and tumor number in the forestomach tissues was recorded. All mice were maintained in specific pathogen free animal facility of Laboratory Animal Center of The First Affiliated Hospital of Nanchang University. All animal experiments were performed with approval from the Institutional Animal Care and Use Committee of The First Affiliated Hospital of Nanchang University.

### Statistical analysis

Data analysis was carried out using GraphPad Prism 8. Statistical significance was calculated by unpaired two-tailed Student’s t-test between two groups or by one-way or two-way ANOVA with Tukey’s corrections when comparing three or more groups. Differences were considered significant when *p* < 0.05. Survival was represented with Kaplan-Meier curves.

## Results

### USP29 plays an oncogenic role in gastric cancer

**Fig. 1:**
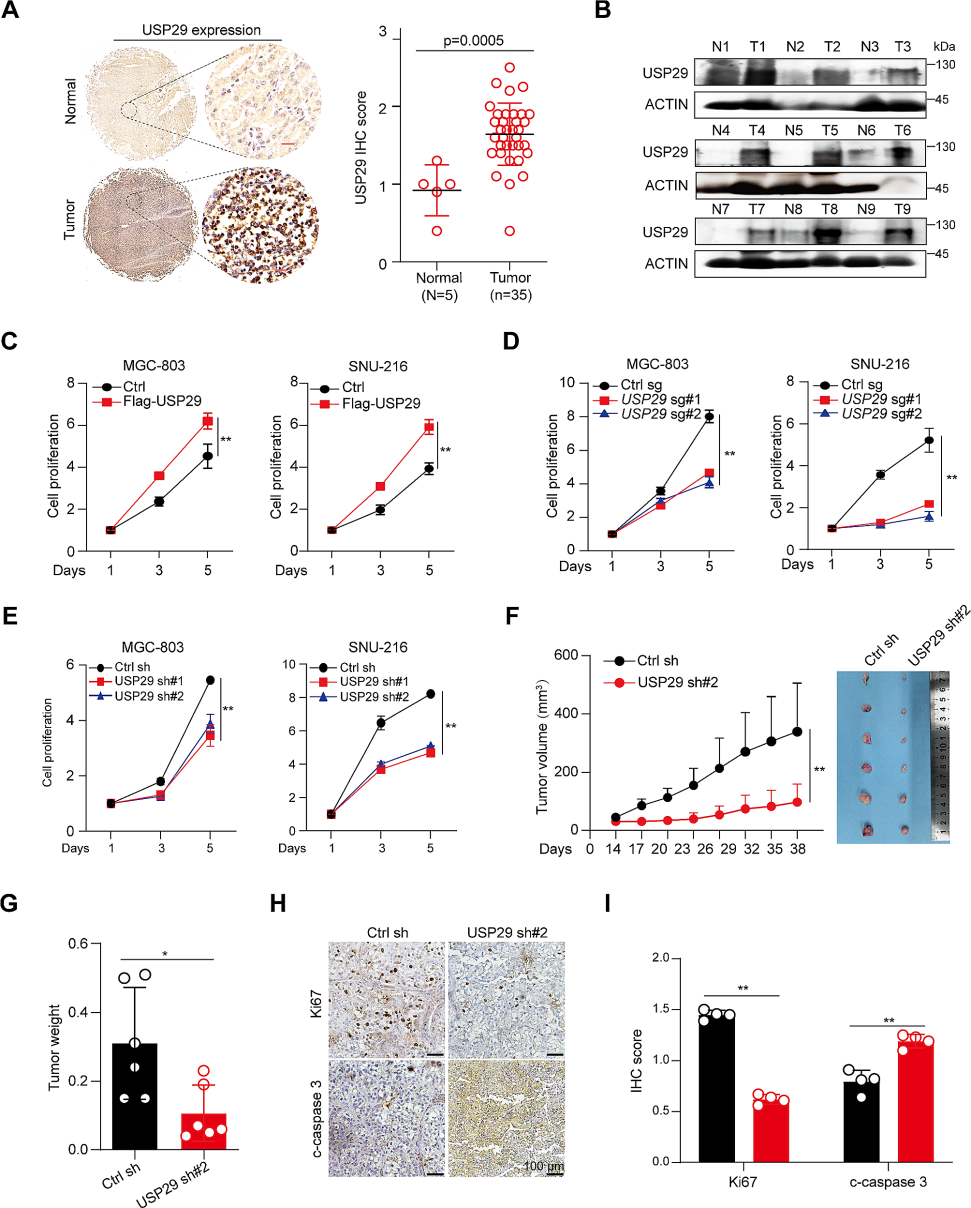
Overexpression and an oncogenic role of USP29 in gastric cancers. (**A**) Left: Representative immunohistological images of USP29 in 5 normal gastric tissues and 35 tumors. Scale bar: 20 μm; right: Quantifications of the immunohistochemistry in the left are shown. (**B**) USP29 protein expression in 9 gastric tumors and paired non-cancerous tissues analyzed using Western blot. (**C**) Cell proliferation of MGC-803 and SNU-216 cells with or without USP29 overexpression. (**D**) Cell proliferation of MGC-803 and SNU-216 cells with or without USP29 depletion by specific sgRNAs. (**E**) Cell proliferation of MGC-803 and SNU-216 cells with or without USP29 knockdown by specific shRNAs. Data shown were obtained from mean ± SD of technical triplicates (**C**-**E**). (**F**) MGC-803 xenograft tumor growth curves and tumor image (*n* = 6). (**G**) Tumor weights of MGC-803 xenografts (*n* = 6). Data shown were obtained from mean ± SD of technical triplicates (**F**, **G**). (**H**) Representative immunohistological images of Ki-67 and cleaved-caspase-3 in MGC-803 xenograft tumors. Scale bar: 100 μm. (**I**) Quantifications of the immunohistochemistry in H. **p < 0.01

To understand the potential role of USP29 in gastric cancer, we first analyzed the USP29 mRNA expression in normal and gastric cancers using the RNA-seq database [29], and revealed that USP29 is markedly overexpressed in the tumor tissues (Supplementary Fig. 1A). We then detected the USP29 protein level in gastric cancer tissue chip (including 5 normal gastric tissues and 35 tumors) using IHC. As shown in Fig. 1A, USP29 was significantly upregulated in gastric cancers. To further verify this finding, nine matched pairs of human gastric cancers and adjacent nontumor tissues were collected for Western blot. The results confirmed the overexpression of USP29 in human gastric cancer tissues (Fig. 1B). Moreover, patients with elevated USP29 mRNA levels showed significantly shorter overall survival compared to patients with lower expression levels of USP29 (Supplementary Fig. 1B).

We next revealed that overexpression of USP29 in gastric cancer cell lines MGC-803 and SNU-216 significantly promoted the tumor cell proliferation (Fig. 1C and Supplementary Fig. 1C). In contrast, depletion of endogenous USP29 by specific sgRNAs or shRNA caused a remarkable downregulation of cell proliferation (Fig. 1D and E, Supplementary Fig. 1C and 1E). We established human gastric cancer xenograft by subcutaneously injection of MGC-803 cells into nude mice. Consisting with the findings obtained in vitro, USP29 knockdown significantly suppressed tumor growth in vivo (Fig. 1F and G). IHC analysis of Ki-67 and cleaved caspase-3 demonstrated strong inhibition of cell proliferation and massive intratumoral cell death in USP29-depleted tumors (Fig. 1H and I). These findings indicated that USP29 may play important roles in gastric cancer initiation and progression.

### USP29 interacts with AURKB and removes its K48-linked polyubiquitination

**Fig. 2:**
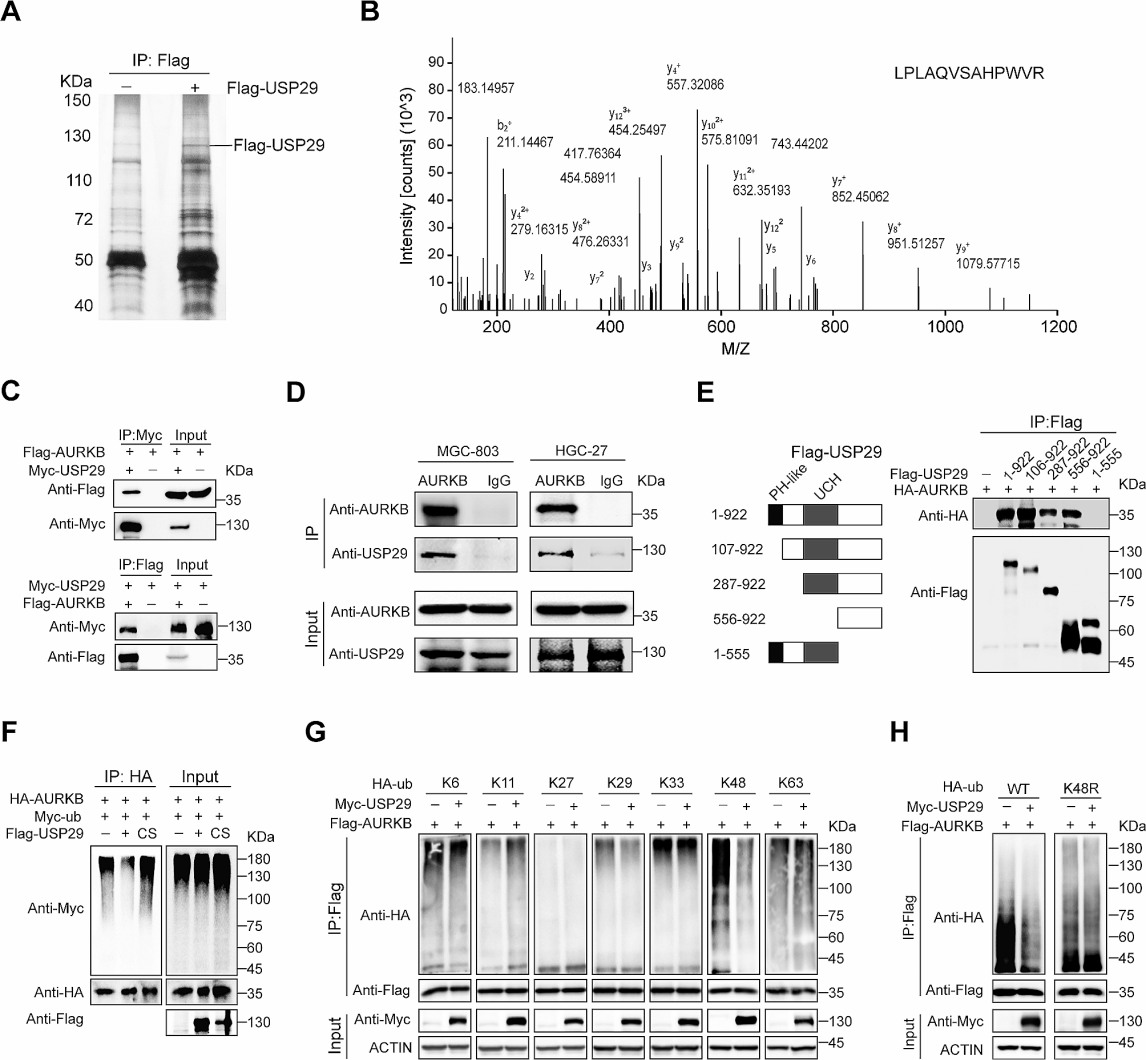
USP29 interacts with AURKB and removes its K48-linked polyubiquitination. (**A**) Immunoprecipitation of Flag-USP29 using anti-Flag antibody. Total cell proteins extracted from MGC-803 cells expressing Flag-tagged USP29 or vector alone were subjected to immunoprecipitation using anti-Flag beads, which were resolved by SDS-PAGE and visualized by silver staining. (**B**) Liquid chromatography-tandem mass spectrometry analysis of the Flag-USP29-associated peptides corresponding to AURKB. (**C**) 293T cells overexpressing Myc-USP29 and/ or Flag-AURKB were subjected to reciprocal Co-IP to detect protein interaction. (**D**) Lysates from MGC-803 and HGC-27 cells were subjected to immunoprecipitation using AURKB antibodies, and USP29 was detected by immunoblot. (**E**) Left: A schematic representation of various Flag-USP29 truncations; right: lysates from 293T cells overexpressing HA-AURKB and respective Flag-USP29 truncation were subjected to Co-IP and immunoblot. (**F**) 293T cells were co-transfected with HA-AURKB, Myc-Ub and Flag-USP29/USP29 C294S mutant (CS), and polyubiquitination analysis of AURKB was shown. **G and H.** 293T cells were co-transfected with indicated Flag-AURKB, Myc-USP29, HA-Ub (WT) and various HA-Ub mutant plasmids, polyubiquitination analysis of AURKB was analyzed. The experiments were independently repeated three times with similar results (A, C-D, right of E, F–H)

As a deubiquitinase, USP29 participates in the regulation of cancer cell fate by affecting its substrates. To identify the binding partners of USP29, we infected 293T cells with vector control and Flag-USP29, respectively, and Flag-bound immunoprecipitates from these cells were resolved by SDS-PAGE and visualized by silver staining (Fig. 2A). The whole lanes were excised and subjected to Liquid Chromatography tandem mass spectrometry analysis. The proteomic data revealed 81 and 39 putative binding proteins (with coverage > 30%) in control and Flag-USP29 immunoprecipitates, respectively (Supplementary Fig. 2A), and AURKB, a well-established oncogenic driver, was specifically immunoprecipitated by USP29 (Fig. 2B and Supplementary Fig. 2B). To validate the mass spectrometry data, we performed reciprocal immunoprecipitation with co-expression of Flag-AURKB and Myc-USP29 in 293T cells and confirmed associations between these both proteins (Fig. 2C). Moreover, co-immunoprecipitation (Co-IP) using MGC-803 and HGC-27 cell lysates validated the specific interaction between endogenous USP29 and AURKB in gastric cancer cells (Fig. 2D). To define the precise region(s) in USP29 for this interaction, we constructed various Flag-USP29 mutants, and expressed a full-length HA-tagged AURKB in combination with respective Flag-USP29 fragments in 293T cells. As shown in Fig. 2E, the carboxyl-terminal region of USP29 (556–922 aa) showed a strong binding with AURKB, whereas the N-terminal (1-555 aa) that contains PH-like and UCH domains exhibited no interaction. These findings demonstrate that AURKB is a bona fide binding partner for USP29.

To assess the potential role of USP29 in regulating AURKB ubiquitination, we co-expressed HA-AURKB, Myc-Ub, and Flag-USP29 in 293T cells, and revealed thatUSP29 markedly impaired the polyubiquitination of AURKB, which requires the DUB activity of USP29, as the catalytic-inactive C294S (substitution of cysteine 294 to serine) mutant that was reported to display a similar substrate-binding capacity [22], failed to reduce AURKB polyubiquitination (Fig. 2F). During formation of the ubiquitin chain, secondary ubiquitin molecules are always linked to one of the seven lysine residues (K6, K11, K27, K29, K33, K48, and K63) or the N-terminal methionine of the previous ubiquitin molecule, and different ubiquitin chain linkages execute distinct cellular functions [30]. To examine which kind of polyubiquitin chains USP29 removes from AURKB, we co-transfected 293T cells with Flag-AURKB, Myc-USP29 and HA-tagged ubiquitin mutants retaining a single lysine residue, and uncovered that USP29 specifically deconjugated the K48-linked polyubiquitin chains (Fig. 2G), a well-established destruction signal to trigger 26 S proteasome-mediated proteolysis [31]. To further explore this notion, Flag-AURKB, Myc-USP29, HA-tagged ubiquitin (WT) or K48R mutant were co-transfected into 293T cells, and the results showed that USP29 failed to catalyze removal of K48R-linked polyubiquitin chains from AURKB (Fig. 2H). These findings imply that USP29 may specifically deconjugate K48-linked AURKB polyubiquitination.

### USP29 enhances AURKB protein stability

**Fig. 3:**
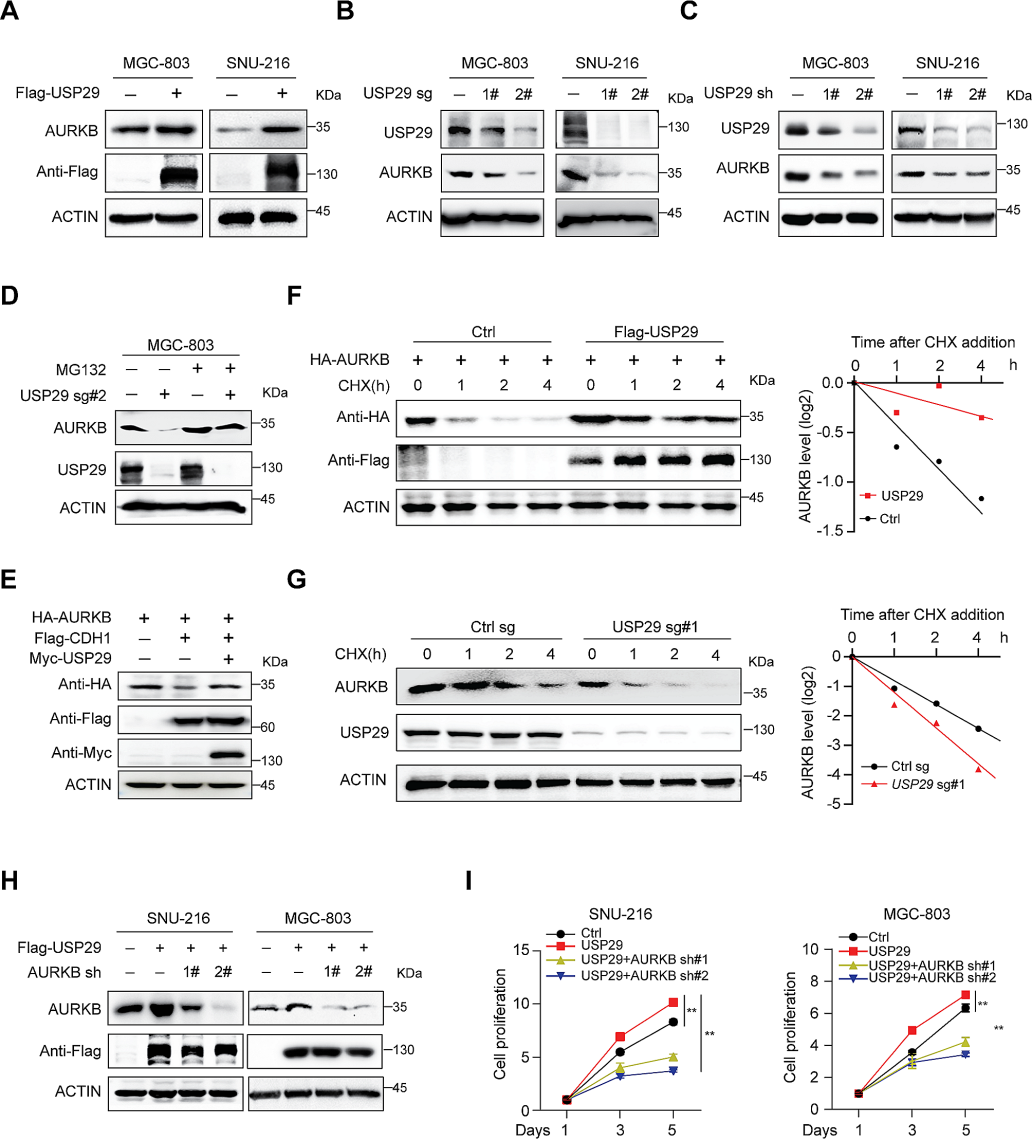
USP29 sustains AURKB protein stability. (**A**) Total cell lysates were extracted from MGC-803 and SNU-216 cells with or without USP29 overexpression, endogenous AURKB level was analyzed by immunoblotting. (**B**) Cells were infected with control or USP29 sgRNAs, USP29 and AURKB protein expression were detected using immunoblot. (**C**) Cells were infected with control or USP29 shRNAs, USP29 and AURKB protein expression were detected using immunoblot. (**D**) MGC-803 cells with or without USP29 depletion were treated with MG132 (10 µM) for 6 h prior to harvest. AURKB and USP29 proteins were analyzed by immunoblot. (**E**) 293T cells were co-transfected with epitope-tagged AURKB, CDH1 and USP29, immunoblot of respective proteins are shown. The experiments were independently repeated three times with similar results (**A**–**E**). (**F**) 293T cells were transfected with indicated plasmids for 24 h, and treated with 100 µg/mL CHX; protein expression was analyzed with immunoblot and protein quantifications are shown. (**G**) MGC-803 cells with or without USP29 depletion were treated with 100 µg/mL CHX and harvested at the indicated time, endogenous AURKB protein were detected by immunoblot and protein quantifications are shown. Data shown were obtained from averages of three independent experiments (**F**, **G**). (**H**) Gastric cancer cells were infected with Flag-USP29 and/or AURKB shRNA viruses, the USP29 overexpression and AURKB knockdown were measured by immunoblots. The experiments were independently repeated three times with similar results. (**I**) Proliferation of stable cell lines in H. Data shown were obtained from mean ± SD of technical triplicates. **p < 0.01

Considering the important role of K48-linked polyubiquitination in protein degradation, we reasoned that USP29 may regulate AURKB protein stability. To test this hypothesis, we ectopically expressed Flag-USP29 in gastric cancer cells and revealed that USP29 overexpression increased the AURKB protein levels (Fig. 3A). On the other hand, depletion of endogenous USP29 using sgRNAs or shRNAs significantly decreased the AURKB protein abundance, but showed minimal effects on AURKB mRNA level (Fig. 3B and C and Supplementary Fig. 3A), indicating that USP29 regulates AURKB expression at the protein level. A previous study reported that AURKB was degraded by the 26 S proteasome via the anaphase-promoting complex/cyclosome (APC/c) and its cofactor CDC20 homolog 1 (CDH1, also known as FZR1) [32]. Here we found that USP29 rescued CDH1-mediated decrease of AURKB and proteasome inhibitor MG132 treatment also rescued the AURKB downregulation induced by USP29 depletion (Fig. 3D and E). To confirm the influence of USP29 on AURKB protein stability, we transfected 293T cells with HA-AURKB and Flag-USP29, and treated the cells with CHX, a widely used inhibitor for protein translation. As expected, overexpression of USP29 extended the AURKB protein half-life (Fig. 3F). Consistently, depletion of endogenous USP29 in MGC-803 cells using sgRNAs significantly accelerated degradation of endogenous AURKB (Fig. 3G).

To examine whether the AURKB protein is an essential downstream effector mediating USP29 oncogenic functions, we depleted endogeneous AURKB expression by shRNAs in SNU-216 and MGC-803 cells, and found that AURKB depletion reverted USP29-overexpression induced growth acceleration (Fig. 3H and I). Consistently, ectopic expression of AURKB also rescued the cell proliferation in USP29 depleted MGC-803 cells (Supplementary Fig. 3B). These data support the notion that USP29 promotes gastric cancer proliferation primarily through stabilization of AURKB.

As deregulation of AURKB is observed in different tumors [4], to test whether USP29 is a global regulator of AURKB, we analyzed AURKB protein expression in neuroblastoma SKN-BE2, lung cancer A549 and colorectal cancer HCT116 cells with or without USP29 depletion. Consistently, USP29 deletion markedly suppressed the AURKB protein abundance in all three cell lines (Supplementary Fig. 3C), arguing that this regulation exists in different types of tumors. Through re-analysis of the RNA seq data using SKN-BE2 and Burkitt’s lymphoma Ramos cells with or without USP29 depletion (GSE180797), we revealed that USP29 depletion resulted in significant downregulation of AURKB pathway (Supplementary Fig. 3D and 3E). These results showed that USP29 is a broad-spectrum regulator of AURKB protein stability.

### Overexpression of FUBP1 directly activates *USP29* transcription in gastric cancers

**Fig. 4:**
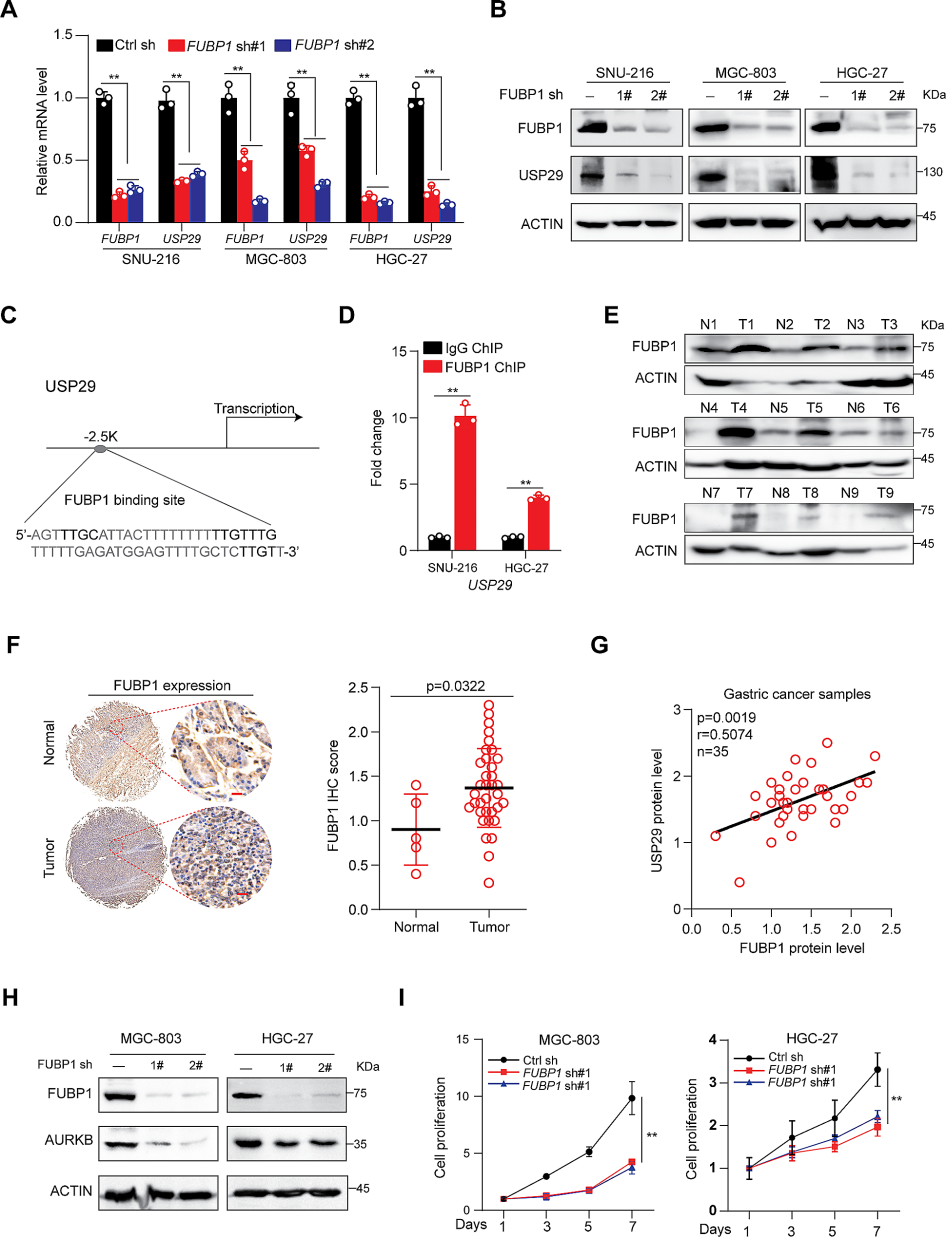
FUBP1 overexpression transcriptionally activates *USP29* in gastric cancers. (**A** and **B**). Gastric cancer cell lines were infected with control or *FUBP1* specific shRNAs, the mRNA (**A**) and protein (**B**) expression of FUBP1 and USP29 were analyzed using qRT-PCR or immunoblot. Graph shows mean ± SD from triplicates; significance was determined by unpaired two-tailed Student’s t-test (**A**). The experiments were independently repeated three times with similar results (**B**). (**C**). A Schematic presentation of potential FUBP1 binding site on the *USP29* promoter. (**D**). ChIP analysis of FUBP1 binding to the *USP29* promoter in SNU-216 and HGC-27 cells. Averages of fold enrichment between the FUBP1 antibodies and IgG were shown. Graph shows mean ± SD from triplicates. (**E**). FUBP1 protein expression in 9 gastric tumors and paired non-cancerous tissues were analyzed using immunoblot. (**F**). left: Representative immunohistological images of FUBP1 in 5 normal gastric tissues and 35 tumors. Scale bar: 20 μm; right: quantifications of the immunohistochemistry are shown. (**G**). Correlation between protein levels of FUBP1 and USP29 in 35 gastric tumors. (**H**). MGC-803 and HGC-27 cells were infected with control or *FUBP1* specific shRNAs, and the expression of FUBP1 and AURKB were analyzed using immunoblots. The experiments were independently repeated three times with similar results. (**I**). Proliferation of MGC-803 and HGC-27 cells with or without FUBP1 knockdown. Data shown were obtained from mean ± SD of technical triplicates. **p < 0.01

We next dissected the mechanisms underlying the upregulation of USP29 in gastric cancers. FUBP1, a multifunctional DNA- and RNA-binding protein, overexpressed in a number of cancers [33]. A previous study suggested that FUBP1 was involved in *USP29* transcriptional activation under oxidative stress (H_2_O_2_ treatment) [34]. However, whether FUBP1 is a bona fide regulator of USP29 in gastric cancers remains unclear. Here, we first depleted FUBP1 expression in gastric cancer cell lines using shRNAs and demonstrated that knockdown of FUBP1 markedly suppressed the gene expression of USP29 in all three cell lines (Fig. 4A and B). A potential FUBP1 binding site was found on the *USP29* transcriptional regulation region (Fig. 4C), and ChIP assay in SNU-216 and HGC-27 cells revealed a significant increase in FUBP1 recruitment to the motif when compared with the immunoglobulin G (IgG) control (Fig. 4D). These data indicated that FUBP1 may directly activate USP29 expression in gastric cancer cells. Moreover, we revealed that FUBP1 protein expression was significantly upregulated in tumor tissues compared to non-cancerous tissues by IHC and immunoblots (Fig. 4E and F). Importantly, there was a significant correlation between FUBP1 and USP29 protein expression levels (*R* = 0.5074, *p* = 0.0019) in 35 gastric cancer samples (Fig. 4G), suggesting that FUBP1 may regulate USP29 expression in vivo.

To investigate the relationship between FUBP1 and AURKB, we analyzed AURKB expression in MGC-803 and HGC-27 cells with or without FUBP1 knockdown, and found that FUBP1 depletion markedly suppressed AURKB protein expression (Fig. 4H), but showed minimal effects on its mRNA level (Supplementary Fig. 4). As expected, FUBP1 depletion also suppressed the proliferation of MGC-803 and HGC-27 cells (Fig. 4I). These results suggest that FUBP1 may promote gastric cancer cell proliferation through a connection of FUBP1-USP29-AURKB axis.

### *Usp29* knockout suppresses BaP-induced gastric carcinogenesis

**Fig. 5:**
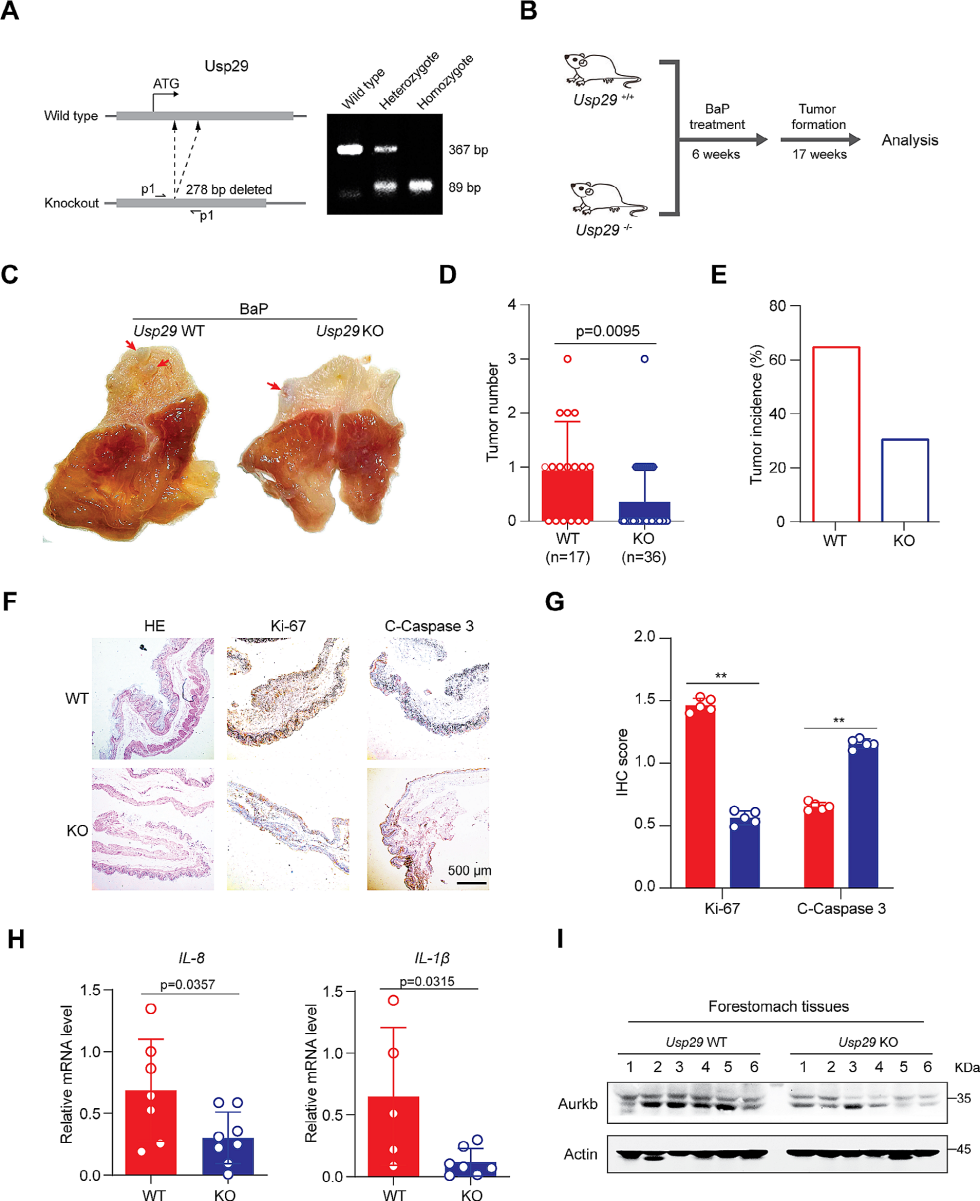
*Usp29* knockout inhibits gastric tumor formation induced by BaP Treatment. (**A**) Left: sgRNA targeting strategy to knockout *Usp29* allele in mice; right: representative genotyping of wildtype and *Usp29* knockout mice. (**B**) Schematic representation of BaP-induced forestomach carcinogenesis. (**C**) Representative images showing gross morphology of stomachs harboring BaP-induced tumors (23 weeks) from wildtype and *Usp29*^*−/−*^ mice. **D and E.** Tumor number (**D**) and tumor incidence (**E**) in stomachs of WT and *Usp29*^*−/−*^ mice after administration of BaP (23 weeks). (**F and G**). Representative images (**F**) and quantification (**G**) of HE, Ki-67, and cleaved caspase-3 staining in WT and *USP29*^*−/−*^ tumors. (**H**). IL-8 and IL-1β mRNA levels in WT and *Usp29*^*−/−*^ stomachs harboring BaP treatment were analyzed by RT-qPCR. Graph shows mean ± SD from triplicates; significance was determined by unpaired two-tailed Student’s t-test. (**I**). AURKB protein expression in WT and *Usp29*^*−/−*^ stomachs harboring BaP treatment were analyzed by immunoblots. **p < 0.01

Carcinogenic intake is one of the key risk factors of inflammation-associated gastric oncogenesis. Benzo[a]pyrene (BaP) is a potent pro-carcinogen employed in initiating cancers, and intragastric administration of BaP was reported to stably induce gastric carcinogenesis in mice [35]. To examine the effects of USP29 on gastric carcinogenesis in vivo, we treated the *Usp29* knockout and wild type mice with BaP, respectively, to induce gastric carcinogenesis (Fig. 5A and B). As expected, the *Usp29* homozygous null mice (*Usp29*^*−/−*^) showed a significant decrease in tumor numbers and tumor incidence upon BaP induction (Fig. 5C, D and E). Pathologic alterations in the forestomach tissues of *Usp29*^*−/−*^ and wildtype mice were examined by HE staining (Fig. 5F). The expression of Ki-67, a cell proliferation marker, was dramatically decreased in the forestomach tissues of *Usp29* null mice, whereas the expression of cleaved caspase-3, a common marker of cell apoptosis, were significantly upregulated (Fig. 5F and G). Furthermore, quantitative analysis of the mRNA levels of IL-1β and IL-8 in gastric tissues showed that these inflammatory cytokines were significantly suppressed in *Usp29*^*−/−*^ mice (Fig. 5H), which was consistent with a previous report that BaP-induced gastric carcinogenesis was accompanied by an elevated inflammatory response [36].Finally, we also revealed that endogenous Aurkb expression was downregulated in *Usp29*^*−/−*^ tissues, suggesting that USP29 may regulate AURKB in vivo (Fig. 5I). Taken together, these findings strongly suggest that USP29 may promote the tumor formation and progression through stabilization of AURKB.

## Discussion

**Fig. 6:**
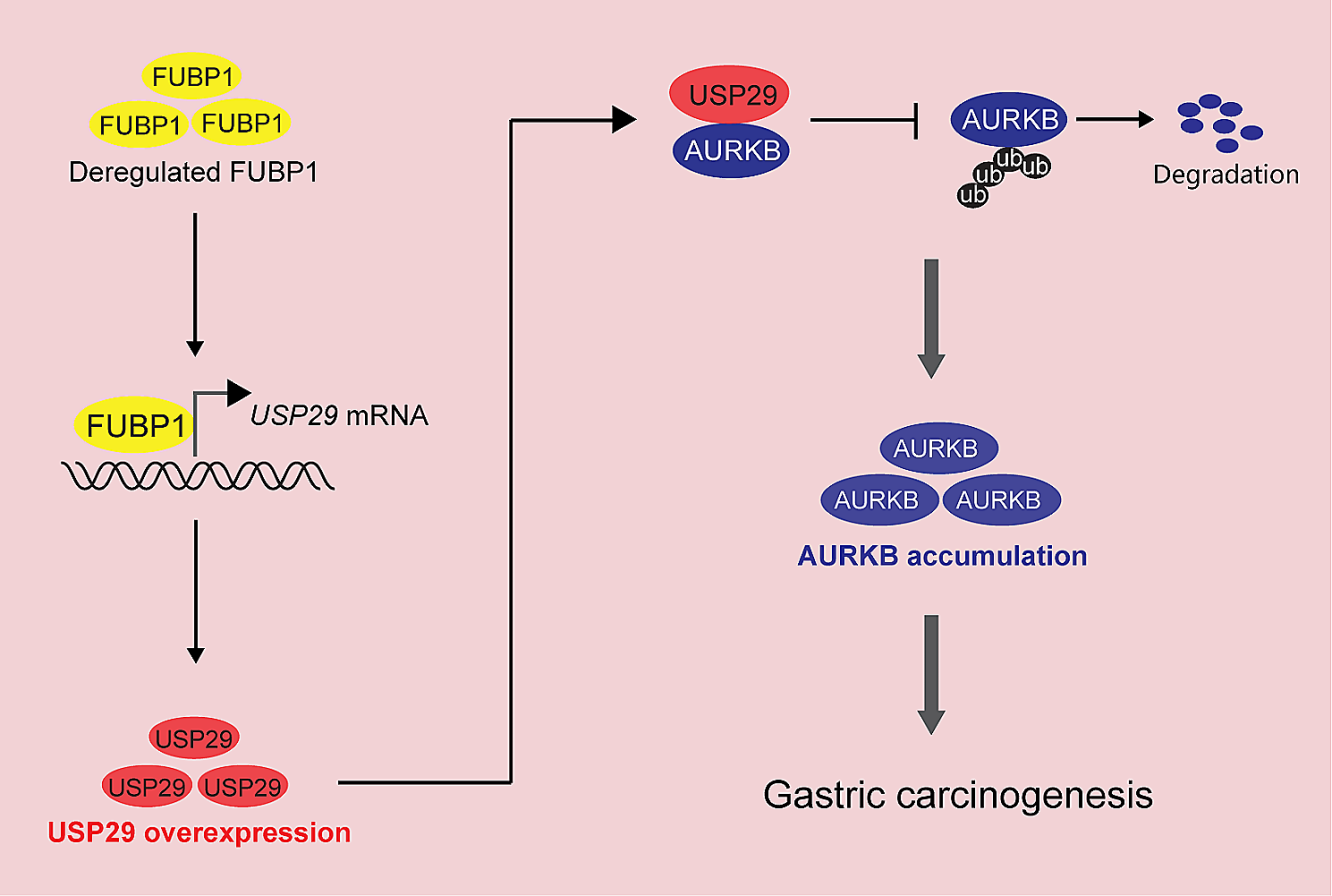
A proposed model depicting regulation of malignant proliferation and tumor progression by the FUBP1-USP29-AURKB axis. Deregulated FUBP1 directly activates USP29 transcription, leading to the overexpression of USP29 in gastric cancer, which subsequently deubiquitinates and stabilizes AURKB, forming a FUBP1-USP29-AURKB regulatory axis. See text for more details

Previous studies have indicated the oncogenic role of USP29 in several cancer types, including the non-small cell lung cancer [37], colorectal cancer [38], hepatocellular carcinoma [23] and triple-negative breast cancer [24]. While USP29 was reported to promote gastric cancer cell migration in vitro, the exact role of USP29 in regulating gastric carcinogenesis remains unknown. In this study, we revealed that USP29 was overexpressed in gastric cancers and promoted tumor cell proliferation. More importantly, deletion of *Usp29* in mice significantly suppressed the carcinogenesis in the BaP-induced gastric cancer model. We also investigated the molecular mechanism and identified a novel FUBP1-USP29-AURKB regulatory axis that enables the oncogenic role of USP29 in gastric cancer (Fig. 6). Our findings demonstrated that USP29 was an important driver of gastric cancer in vivo, and uncovered a key molecular mechanism underlying gastric cancer progression.

Previous studies reported that AURKB overexpressed in gastric cancer [39] and promoted several malignant phenotypes including cell proliferation [10], metastasis [9] and drug resistance [12, 40]. However, the upstream regulatory mechanism of AURKB expression in cancers remains unclear. Through a mass spectrometry analysis of USP29 binding proteins, we identified USP29 as a novel AURKB deubiquitinase, which specifically deconjugates its K48-linked polyubiquitination and is crucial to AURKB stabilization in gastric cancer cells. Considering the important role of AURKB in controlling cell cycle, we reasoned that USP29 may promote gastric cancer cell proliferation by regulating cell cycle progression. Indeed, our findings, along with previous reports that depletion of USP29 caused cell cycle arrest and apoptosis in cervical cancer [25], colorectal cancer [38], and osteosarcoma cell [41]. As well as AURKB protein stability was regulated by USP35 [18], USP13 [19] and USP48 [20], indicate that deubiquitination of AURKB may be essential to its oncogenic activity.

Targeting deubiquitinases (DUBs) has been recognized as a potential strategy for cancer treatment [17, 42–44]. However, it remains unclear which DUB may represent an ideal and realistic therapeutic target. We found that USP29 stabilizes AURKB in gastric cancer, neuroblastoma, lung cancer and colorectal cancer. Similar to other USP29 substrates c-MYC, HIF1α and Snail1, AURKB deregulation often occurs in cancers, implying that USP29 may become a potential target for cancer treatment. Given the fact that *Usp29* knockout mice are viable without noticeable defects, suggesting that USP29 is dispensable for normal development and physiological functions [22], and specific USP29 inhibitors are likely to cause minimal side effects in cancer treatment.

## Conclusions

In conclusion, this study identified a novel FUBP1-USP29-AURKB regulatory axis that may play important roles in gastric carcinogenesis and tumor progression. Our findings may provide rationales for development of therapeutic strategies for gastric cancers by targeting USP29.

### Electronic supplementary material

Below is the link to the electronic supplementary material.

Supplementary Material 1

## Data Availability

No datasets were generated or analysed during the current study.

## Abbreviations

APC/c: Anaphase promoting complex/cyclosome
AURKA: Aurora kinase A
AURKB: Aurora kinase B
AURKC: Aurora kinase C
BaP: Benzo[a]pyrene
BOP1: BOP1 ribosomal biogenesis factor
CDC25A: Cell division cycle 25 A
CDH1/FZR1: CDC20 homolog 1
CDK1: Cyclin-dependent kinase 1
ChIP: Chromatin immunoprecipitation
CHX: Cycloheximide
Co-IP: Co-immunoprecipitation
FBS: Fetal bovine serum
FUBP1: Far upstream element binding protein 1
IHC: Immunohistochemistry
IgG: Immunoglobulin G
qRT-PCR: Quantitative real-time PCR
SDS-PAGE: SDS polyacrylamide gel
TBS: Tris-buffered saline
TBST: TBS containing 0.1% Tween 20
TWIST1: Twist family bHLH transcription factor 1
USP: Ubiquitin-specific peptidase
