# Supplementary material for: USP29 activation mediated by FUBP1 promotes AURKB stability and oncogenic functions in gastric cancer
Source: Cancer Cell Int. 2024 Jan 17;24:33. doi: 10.1186/s12935-024-03224-5 (PMC10792871; doi:10.1186/s12935-024-03224-5)
Supplement: Supplementary file 1 — Supplementary Material 1 [file 12935_2024_3224_MOESM1_ESM.docx]

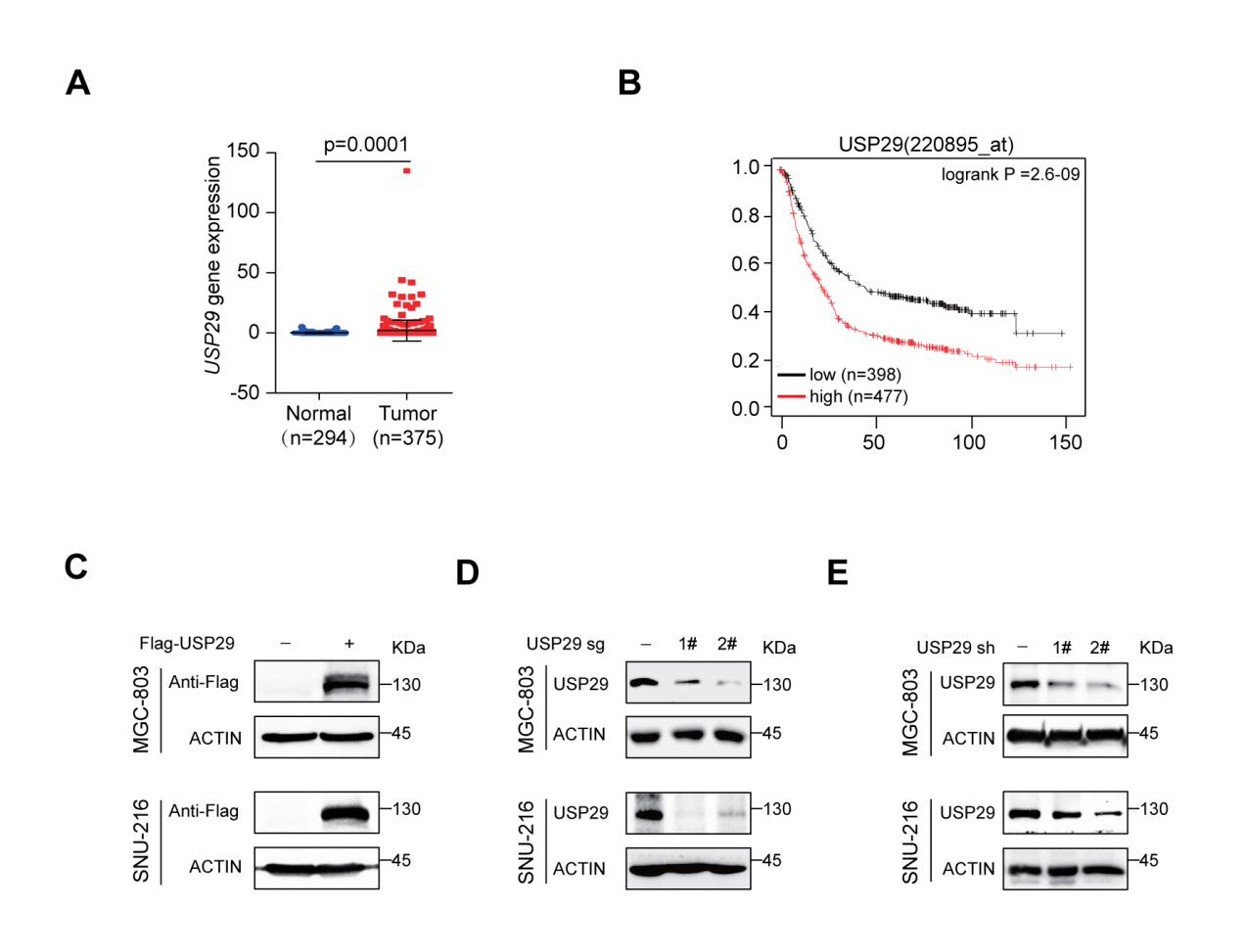


**Supplementary Figure 1. Related to Fig. 1**

**A.** Relative USP29 mRNA expression in normal gastric tissues and gastric cancers (<http://kmplot.com/analysis/>). **B**. Kaplan–Meier survival curves of gastric cancer patients based on *USP29* expression (<http://kmplot.com>). **C-E**. Immunoblots showing the overexpression or depletion of USP29 in gastric cancer cell lines. The experiments were independently repeated three times with similar results.


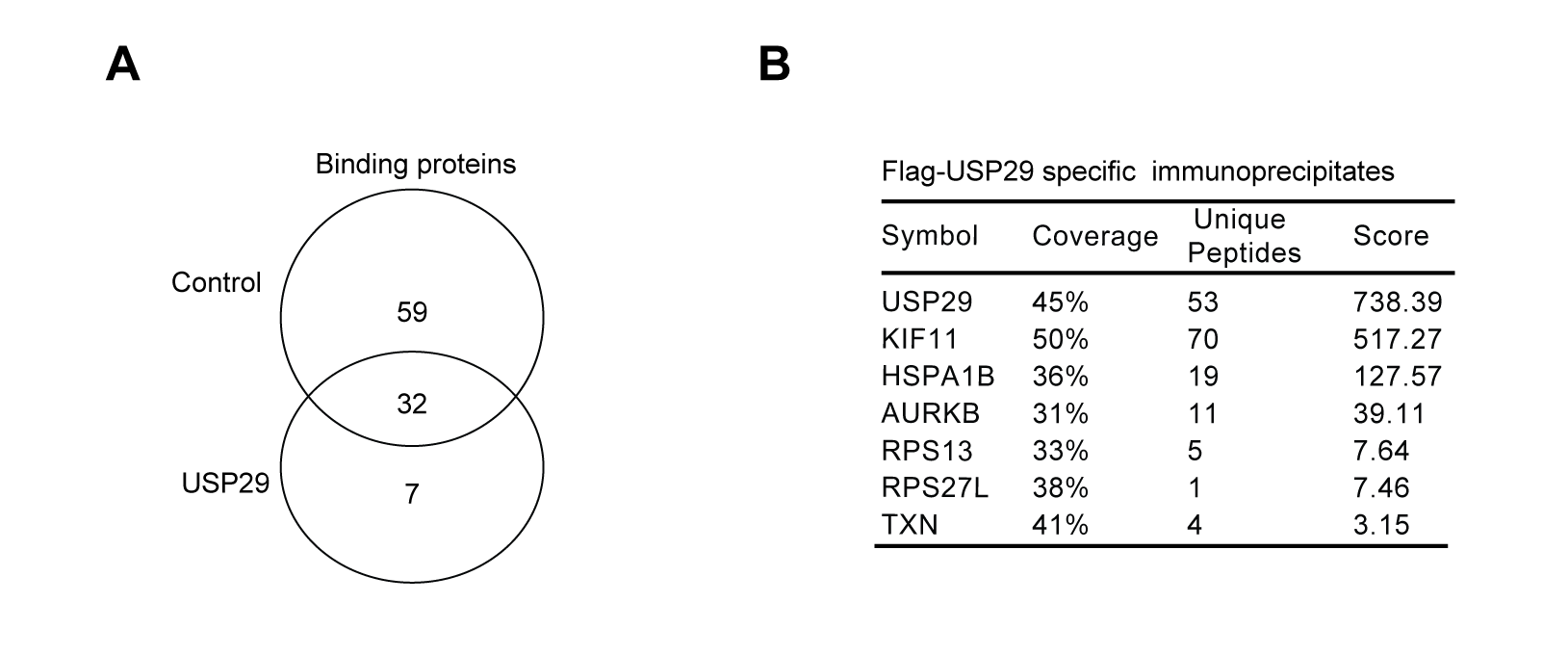


**Supplementary Figure 2. USP29 interacts with AURKB.**

**A**. The Venn diagram showing the binding proteins co-precipitated in 293T cells with or without Flag-USP29 expression. 91 and 39 proteins (with coverage>30%) were detected in control and Flag-USP29 overexpressed cells, respectively, and 32 proteins were overlapped in both cells. **B**. Seven candidates specifically immunoprecipitated in Flag-USP29 expressing cells were listed.


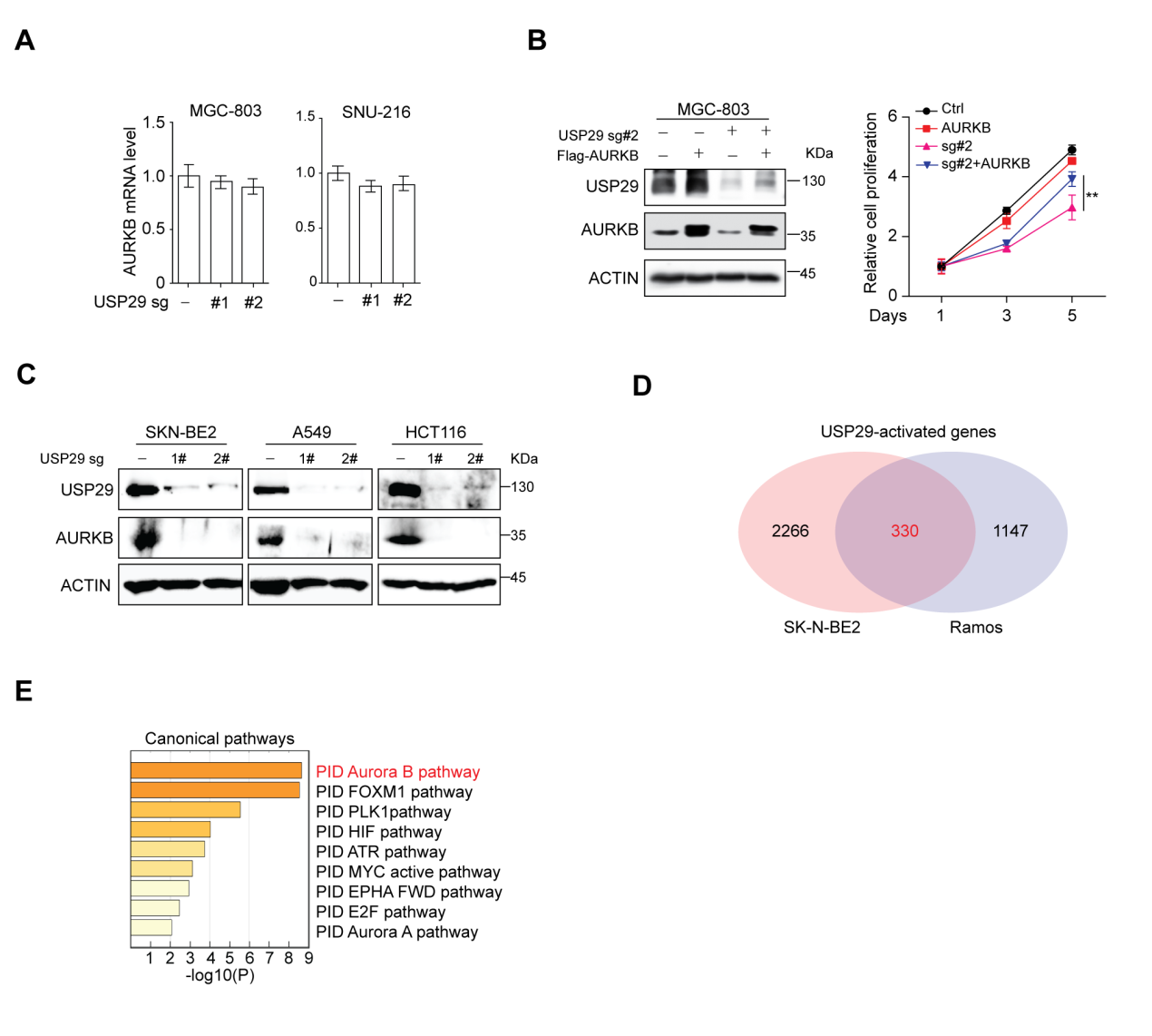


**Supplementary Figure 3. USP29 depletion decreases AURKB protein abundance.**

**A**. Cells were infected with control or USP29 sgRNAs and total mRNA was extracted, USP29 mRNA level was analyzed using qRT-PCR. **B**. Proliferation of MGC-803 cells upon USP29 depletion by sgRNA along with ectopic expression AURKB. **C**. Cells were infected with control or USP29 sgRNAs and total protein was extracted, USP29 and AURKB protein levels were analyzed by immunoblot. **D**. Venn diagram of downregulated genes resulting from USP29 depletion in SK-N-BE2 and Ramos cells. Downregulated genes were identified as those with p<0.05, and FPKM>1 using edgeR software, GSE180797. **E**. Pathway enrichment analysis of 330 overlapped genes using Canonical Pathways database (<https://metascape.org>). The experiments were independently repeated three times with similar results (A,B and C). Data shown were obtained from mean±SD of technical triplicates (right of E). **p<0.01.


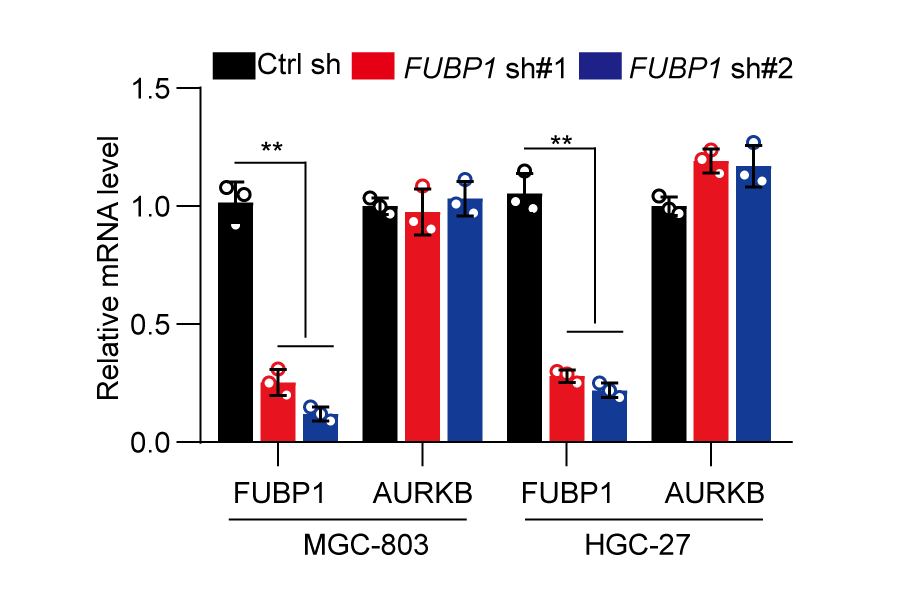


**Supplementary Figure 4. FUBP1 knockdown shows minimal effect on AURKB mRNA expression.** MGC-803 and HGC-27 cells were infected with ctrl or FUBP1 shRNAs, the mRNA level of FUBP1 and AURKB were analyzed by qRT-PCR. Graph shows mean±SD from triplicates; significance was determined by unpaired two-tailed Student’s t-test. **p<0.01.

**Supplementary Table S1: Primers used in this study**

| **rimers for qRT-PCR** |  |
| --- | --- |
| *USP29* forward | 5^’^-GAAACTCGGGCCTTCATTCAA-3^’^ |
| *USP29* reverse | 5^’^-CTGTGCTCTGGTTCCAATGG-3^’^ |
| *FUBP1* forward | 5^’^-CAACCAGATGCTAAGAAAGTTGC-3^’^ |
| *FUBP1* reverse | 5^’^-CCTCCTCTGCCAATTATGAATCC-3^’^ |
| *ACTIN* forward | 5^’^-CATGTACGTTGCTATCCAGGC-3^’^ |
| *ACTIN* reverse | 5^’^-CTCCTTAATGTCACGCACGAT-3^’^ |
| *AURKB* forward | 5-ATCAGCTGCGCAGAGAGATCGAAA-3′ |
| *AURKB* reverse | 5-CTGCTCGTCAAATGTGCAGCTCTT-3′ |
| **sgRNAs** | |
| *USP29* sg#1 | 5’-AATTTGGGGAGCTCCCGATA-3’ |
| *USP29* sg#2 | 5’-TTGAGCAGTAGTGCACCTGT-3’ |
| Ctrl sgRNA | 5’-GGGCGAGGAGCTGTTCACCG-3’ |
| **shRNAs** | |
| *FUBP1 sh#1* | 5’-ACTACTGATAGGAGGTTAATA-3’ |
| *FUBP1 sh#2* | 5’-TACAACCCTGCACCTTATAAT-3’ |
| *USP29* sh#1 | 5′‐TGTGTGGAGTATCTTGGTGTA‐3′ |
| *USP29 sh#2* | 5′‐CTGGTGAAGAATAACGAGCAA‐3′ |
| Ctrl shRNA | 5’-TACAACAGCCACAACGTCTAT-3’ |
| **Primers for CHIP** | |
| ACTIN promoter forward | 5'-GACTTCTAAGTGGCCGCAAG-3' |
| ACTIN promoter reverse | 5'-TTGCCGACTTCAGAGCAAC-3' |
| USP29 CHIP forward | 5'-AGGAGGTGGAGGTTGCAGTGA-3' |
| USP29 CHIP forward | 5'-TGCTTCCCAAGGACTGCTCCT-3' |
